# The Influence of Alkali Metals on the Doping of Poly(*p*-phenylene) Oligomers

**DOI:** 10.3390/molecules27248699

**Published:** 2022-12-08

**Authors:** Laura Oliveira Péres, Rebeca da Rochas Rodrigues, Guy Louarn

**Affiliations:** 1 Department of Exact and Earth Sciences, Campus Diadema, Federal University of São Paulo (UNIFESP), São Paulo 09913-030, Brazil; 2 Institut des Matériaux de Nantes Jean Rouxel (IMN), CNRS, Nantes Université, F-44000 Nantes, France

**Keywords:** phenylene, oligomers, X-ray, Raman, terphenyl, quaterphenyl

## Abstract

In this study, we report on crystallographic studies that were performed on Na- and K-doped terphenyl and quaterphenyl. The data obtained via X-ray scattering and transmission electron diffraction show that, for both K-doped terphenyl and quaterphenyl samples, there is an increase in the *c* parameter. However, in regard to Na-doped terphenyl, there is a *c* parameter decrease along with an *a* parameter increase, which may be accounted for by the polymerization of this oligomer. Moreover, in order to complete the crystallographic study, a Raman analysis was conducted to describe the localization of the radical anions and the local distortions induced by the electric charges during the doping process.

## 1. Introduction

It has been shown that organic optoelectronic devices are a key issue in plastic electronics [1]. From the period in which Burroughes et al. [2] demonstrated that a green-yellow light emission could be achieved in organic light-emitting diodes (OLEDs), improvements have been made in producing blue emission devices, as well as in devices with high quantum efficiency [1]. Conjugated polymers, such as poly(*p*-phenylene)s (PPP), become highly conductive when doped with electron donors or acceptors [3]. Moreover, there are important materials for the purposes of development for devices such as solar cells [4], OLEDs [5], and sensors [6,7]. One of the most sensitive points that requires clarification in regard to the doping of conjugated polymers is what happens in the charge transfer process between the polymer chains and the counter-ions. These materials can be oxidized or reduced, chemically or electrochemically, thereby promoting a high conducting state. This is also the reason why certain reports have already appeared and have provided interesting information on this point [8,9,10,11]. Moreover, some of them have reported on the oligomer’s behavior, due to the fact that they may be studied as model compounds of the corresponding polymer.

In this respect, the alkali metal doping of poly(*p*-phenylene) (PPP), and its oligomers, has already been studied via in situ FT Raman and electron spin resonance (ESR) techniques [11]. Spectroscopy techniques are extensively used for the purposes of determining the characterization of materials with different metals [12,13,14]. Specifically, in the case of Fung et al. [11], the authors demonstrated that Raman and ESR are complementary. They demonstrated the coexistence of polarons and bipolarons in the early stages of the intercalation process. This was in addition to the heterogeneous distribution of the dopant ions in the polymer electrode. Further, they showed that in a composition of Na_0.5_(C_6_H_4_), bipolarons are the main structural defects.

Crystallographic studies are another way to study the doping of conjugated polymers. In the work developed by Dubois et al. [3], PPP was intercalated with sodium and lithium, i.e., two metal alkali atoms with a small size. The results showed a reduction in the crystallinity of the doped polymer (at 30%) and the process was explained through a two-step mechanism: the intercalation of the alkaline ions into the crystalline domains and the insertion into the amorphous regions of the polymer.

In regard to the PPP oligomers, which are completely crystalline (especially in regard to *p*-sexiphenyl), certain studies were conducted in order to understand the metal intercalation [15,16]. In all cases, a maximum of 0.5 metal/phenyl and a well-ordered structure were observed. Furthermore, the material obtained was heterogeneous due to the fact that several phases coexist.

In the present work, we present the results of Raman spectroscopy, X-ray, and electron diffraction measurements in order to further understand the doping process of *p*-terphenyl and *p*-quaterphenyl reduced (doped) with alkali metal (Na and K) donor ions, (which show very different ionic radii). In particular, through Raman spectroscopy, we also investigated the nature and the location of the formed radical anions. This provided us with a better understanding of the charge storage configurations, which is crucial for any prediction of their ability to form high-charge states.

## 2. Results and Discussion

### 2.1. Analysis of Electron and X-ray Diffraction

#### 2.1.1. Pristine Materials

Prior to the sodium or potassium doping, the structure of our quaterphenyl pristine material was investigated for the purposes of comparison (see Figures S1 and S2 in the Supplementary Materials) (in an attempt to find similarities to those that have been found in the literature [17]). The structure refinement was conducted in the P2_1_/a space group, using the Rietveld method with cell parameters *a* = 8.0921(3) Å, *b* = 5.6032(2) Å, *c* = 17.8524(13) Å, and β = 95.702(5) for *a* = 8.110(6) Å, *b* = 5.610(4) Å, *c* = 17.910(10) Å, and β = 95.80(6), respectively (as in the paper mentioned above). One has to notice a typographical mistake in one of the atomic positions given in this specific paper: the C(8) y position has to be read as a positive value. The quality of the Rietveld refinement is χ^2^ = 3.4 and R_wp_ = 6.1. The cell parameters found by conducting the electron diffraction were in good agreement with those determined via X-ray.

For the purposes of the terphenyl pristine material, the cell parameters obtained in the P2_1_/a space group were found to be: *a* = 8.1041(5) Å, *b* = 5.6123(4) Å, *c* = 13.6207(11) Å and β = 91.957(7) for *a* = 8.019(2) Å, *b* = 5.576(2) Å, *c* = 13.578(2) Å, and β = 92.10(2), according to the literature [18]. The figures of merit for this refinement were χ^2^ = 8.69 and R_wp_ = 7.36. We have no explanation for the discrepancy between the *a* parameters in the two cases, which were found to be three times larger than the level of standard uncertainty. However, the atom positions were in good agreement with the published results and the *a* cell parameter was the same in our pristine material, as well as in the sodium- or potassium-doped compounds that also contain the non-doped terphenyl phase.

In regard to all the patterns of doped-material presented below, the high inhomogeneity of the samples requires mentioning. Certain crystallites were non-doped, whereas others exhibited certain differences in cell parameters, which could be related to doping effects. In certain cases, impurities could also be detected. Most often, only certain line shifts could be attributed to *c* parameter values, due to the too ill-defined X-ray patterns. Therefore, electron diffraction experiments are very useful in that they can help with separately studying the different phases and impurities, which can then be utilized to confirm the cell parameters found via X-ray diffraction.

#### 2.1.2. Sodium-Doped Material

Figure S3 of the Supplementary Materials demonstrates the comparison of both pure quaterphenyl and Na-doped material. It is mainly only the pattern, where the background was removed for clarity reasons, which remains the same as that of pure quaterphenyl (with the same peak positions). Although, having said this, the NaNH_2_ impurity is highlighted. In addition to this impurity, certain extra peaks can be observed. These extra peaks are located at 11.60, 15.57, and 15.81 degrees. Moreover, 2 theta can be indexed respectively as (−1 0 1), (1 0 2), and (0 1 0), if one were to assume the removal of both the 2_1_ screw axis and the a glide plane from the starting P2_1_/a space group. This is quite surprising, due to the fact that this space group change can only be due to some atomic rearrangement in the material. Moreover, this modification is usually accompanied by certain cell parameter variations. However, the information provided by such a complicated pattern are too poor to proceed with and the hypothesis mentioned here can explain all the pattern features.

**Figure 1:**
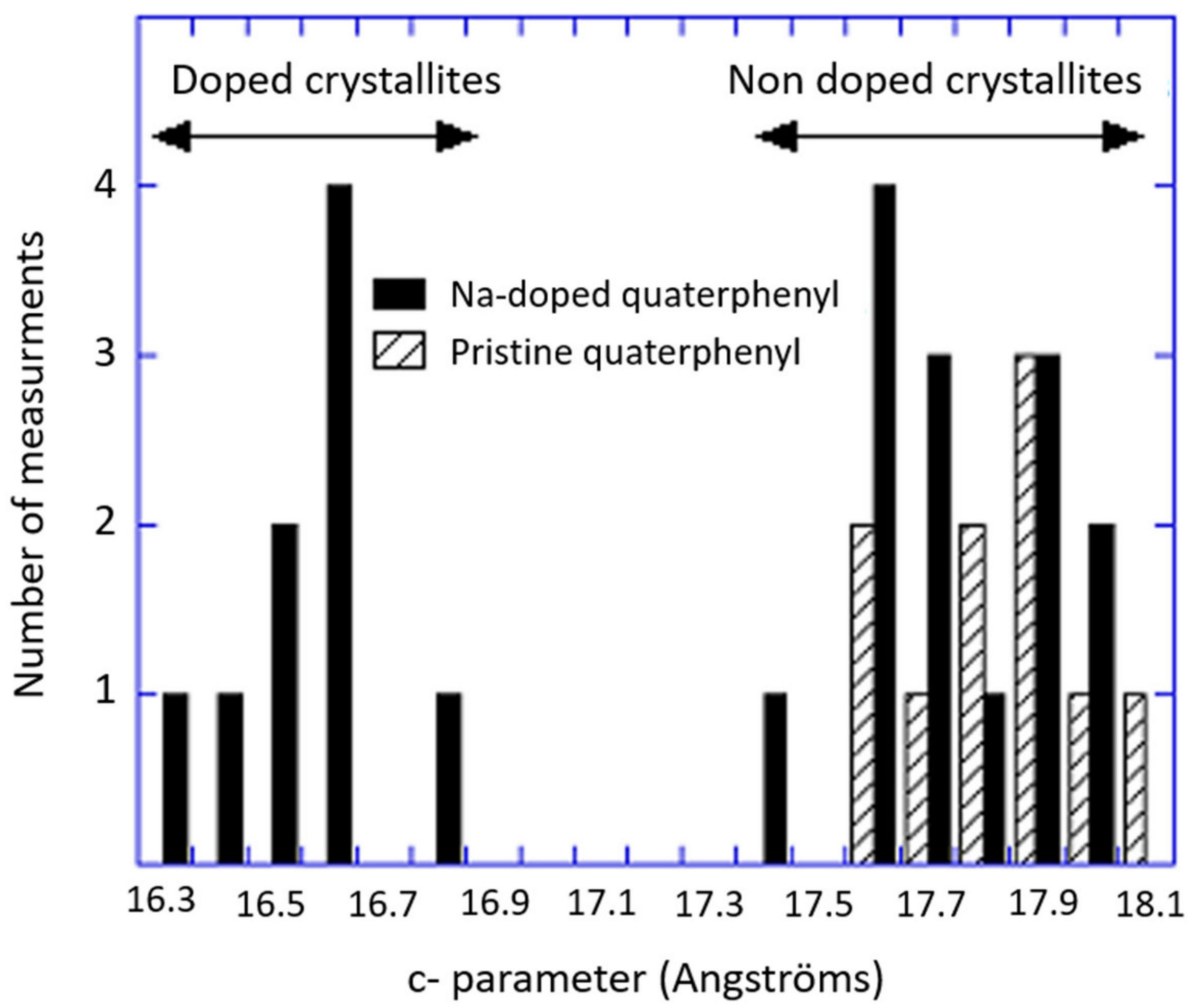
The comparative histograms of the pristine and Na-doped quaterphenyl.

NMR and electron diffraction confirm the presence of NaNH_2_. Both of these techniques show the existence of non-doped materials and NMR indicates the occurrence of two different Na-doped phases [19]. As is shown in Figure 1, the histogram of the *c* parameter—obtained via electron diffraction that exhibits two contributions—one centered at 17.8 Å (due to non-doped crystallites) and the other one at 16.5 Å, which has to be correlated to a Na-doped phase that was not observed in the X ray pattern. This was most likely due to the fact that a possessed a too short coherence length in regard to the particles.

**Figure 2:**
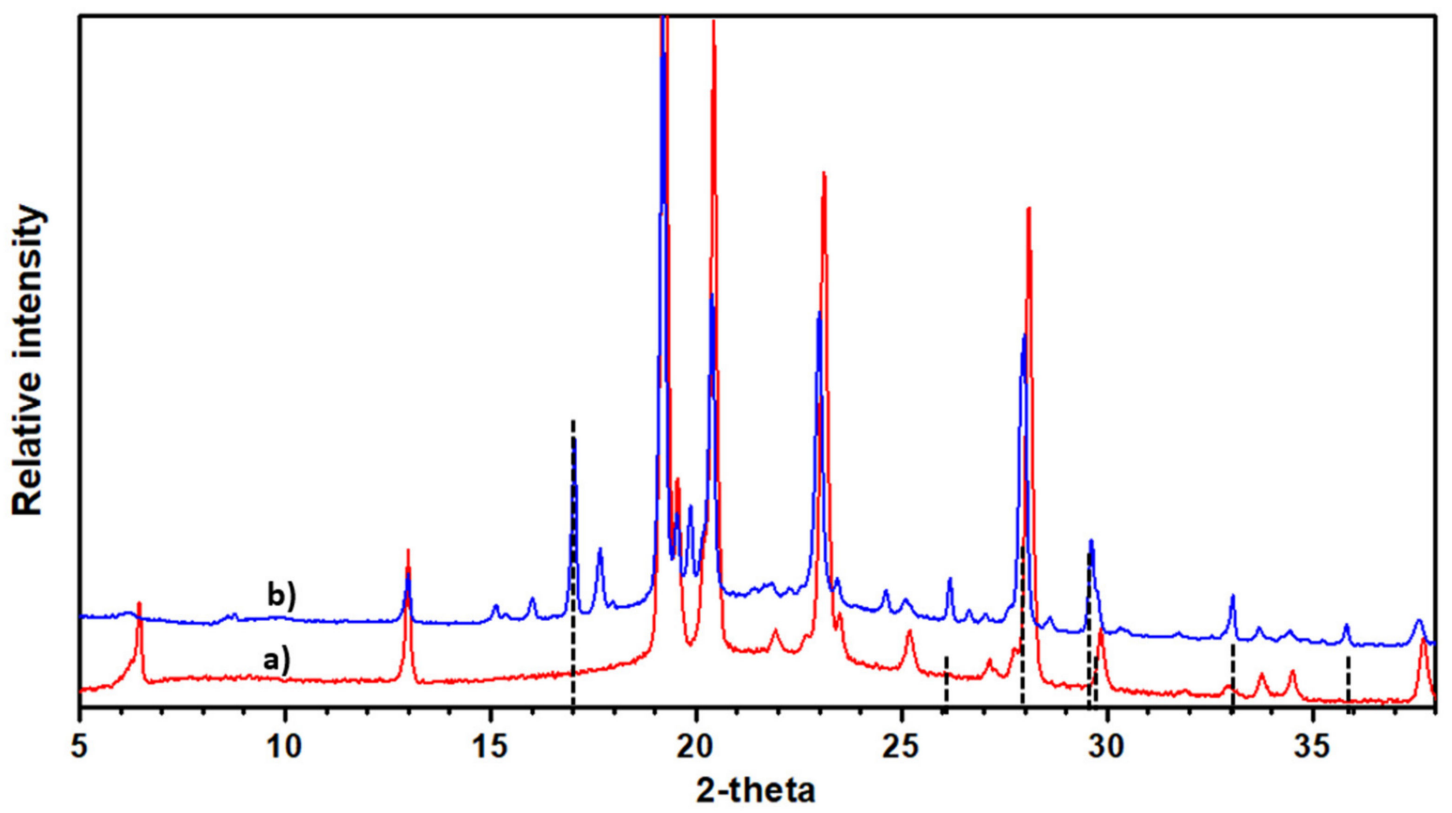
The ϕ3-Na pattern (spectrum **a**) when compared with the pristine material (spectrum **b**). NaNH_2_ impurity is indicated as vertical markers. Lines are indexed according to the space group and the cell parameters given in the text for the Na-doped terphenyl.

In regard to the Na-doped terphenyl, the XRD pattern shows the occurrence of NaNH_2_ as an impurity and a remaining non-doped terphenyl phase, as can be seen in Figure 2. In addition, certain small extra peaks can be found and indexed as reported in Table 1, using the following monoclinic cell parameters: *a* = 15.403(3) Å, *b* = 6.089(13) Å, *c* = 17.84(5) Å, and β = 89(1).

This hkl attribution was obtained thanks to Crystal Cracker software (Version 189, Center Arizona State University, USA). The obtained cell parameters were subsequently refined using the Chekcell package. The systematic extinction is compatible with the possible space groups: P2_1_ and P2_1_/m. Due to the poor quality of the pattern and the polyphased nature of the compound, this unit cell determination has to be taken cautiously as different Na-doped compounds may coexist (as observed in the case of the potassium-doped quaterphenyl). In particular, a peak remains unexplained around 8.77° 2θ. Unfortunately, no electron diffraction data were available for this compound in order to confirm the results presented above.

#### 2.1.3. Potassium-Doped Materials

**Figure 3:**
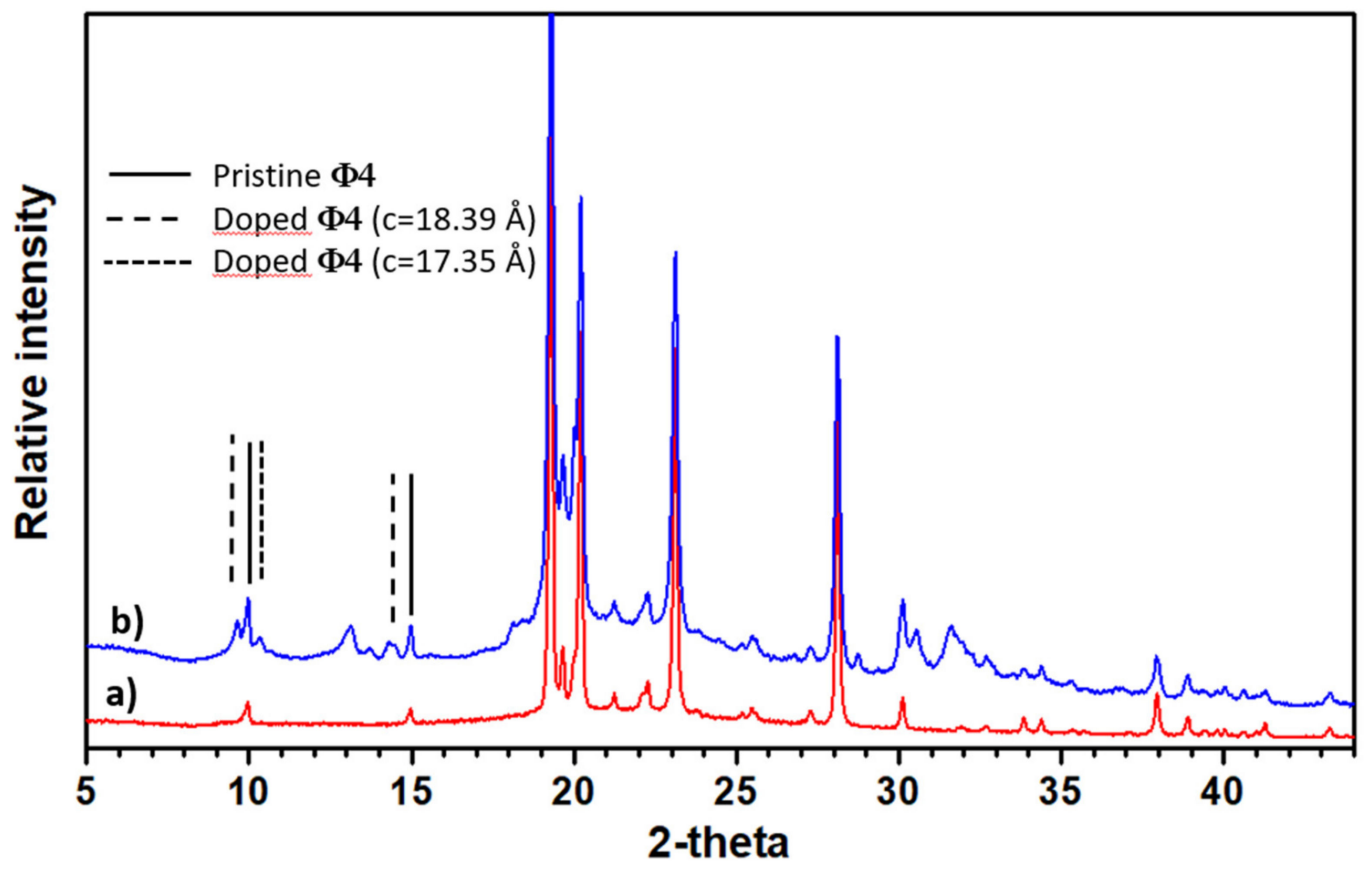
The ϕ4-K pattern (spectrum **a**) compared with the pristine material (spectrum **b**). The (00l) lines are indicated in the former in regard to the different *c* parameter values.

**Figure 4:**
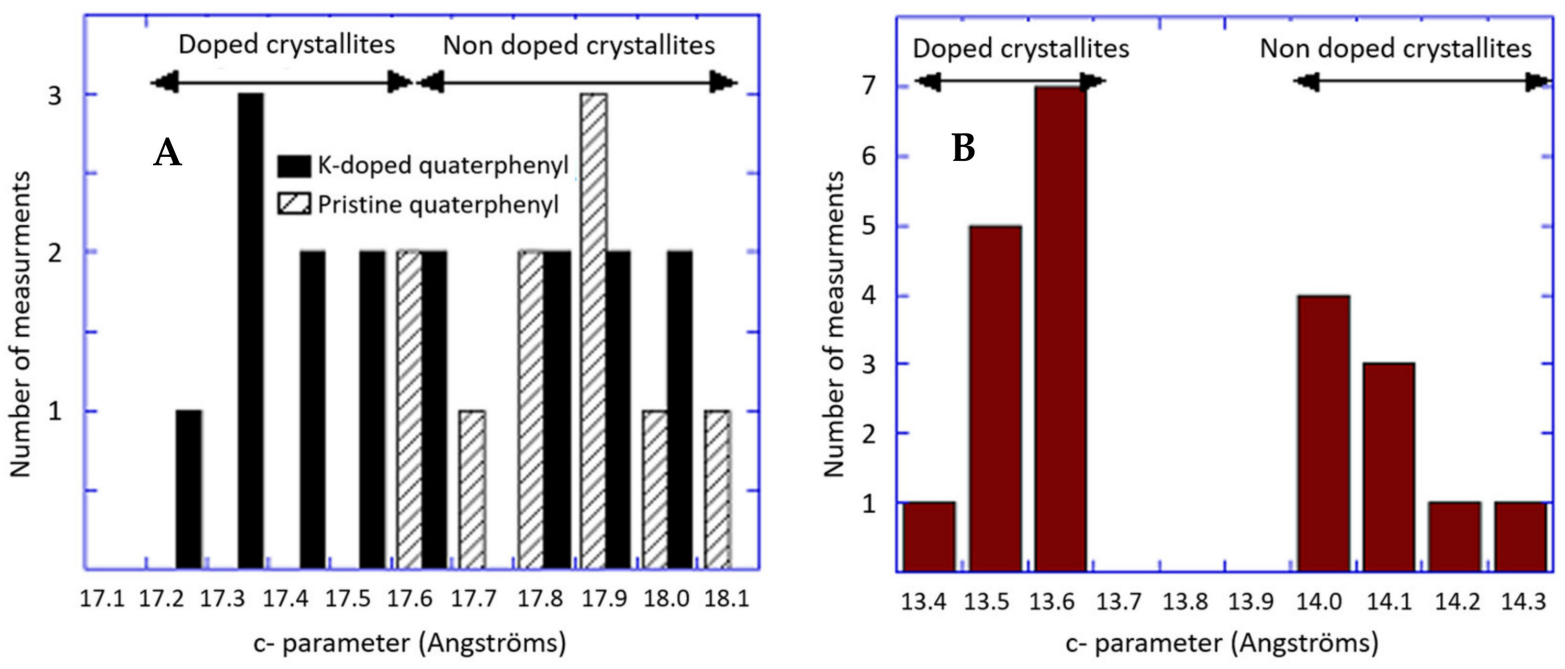
The comparative histograms of the pristine and K-doped quaterphenyl (**A**). Histogram of the K-doped terphenyl (**B**).

In contrast to the Na-doped quaterphenyl, no occurrence of KNH_2_—as an impurity—is observed in the X-ray pattern (see Figure 3). This can be fully explained by the coexistence of the pristine material and a potassium-doped phase with an 18.39 Å *c* parameter (when there is an increase of 0.5 Å of the quaterphenyl *c* parameter), whereby there is a giving of two extra peaks at 9.67 and 14.50 degree 2 theta, indexed respectively, as (0 0 2) and (0 0 3). A small additional peak (2 theta = 10.31°) provides a *c* parameter of approximately 17.35 Å c (i.e., a decrease of 0.5 Å of the quaterphenyl *c* parameter), which is in good agreement with the *c* parameter value obtained from an electron diffraction on a single crystal (see Figure 4A). The remaining question that should be investigated is as follows: what is the reason why there are no crystals with an 18.39 Å c observed in the electron diffraction experiments? In this regard, the only possible explanation is a problem of sampling. Indeed, the two sets of *c* parameters, as observed from the XRD analysis, prove the inhomogeneity of the sample.

**Figure 5:**
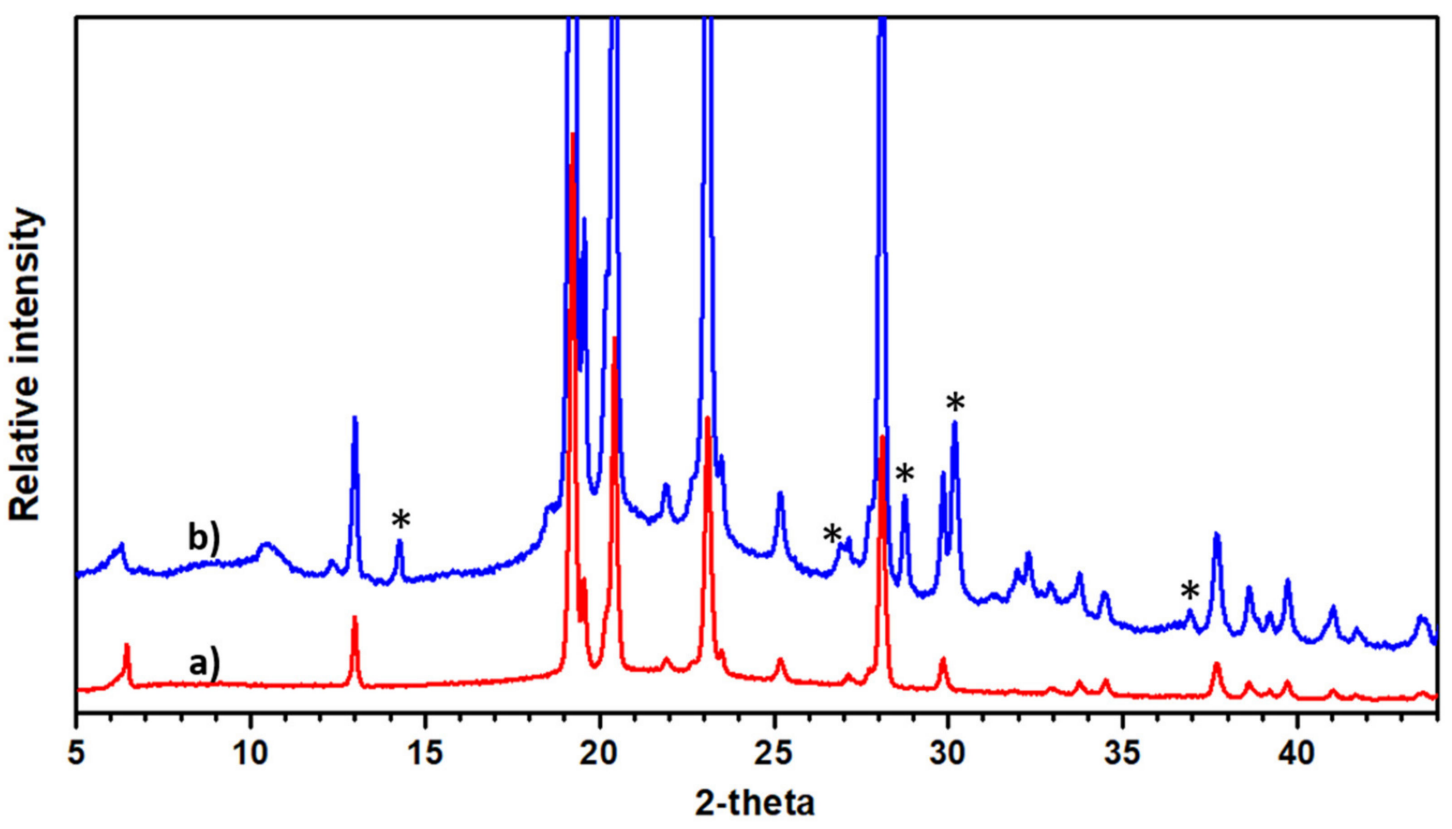
The ϕ3-K pattern (**a**) compared with the pristine material (**b**). KNH_2_ impurity is indicated with asterisks. Extra peaks can be attributed to (00l) lines with a *c* parameter value of 14.34 Å.

In regard to the K-doped terphenyl, KNH_2_ phase impurity is observed (see Figure 5). In the main, all the peaks of the pristine material remain unchanged, indicating that a part of the terphenyl is not doped. However, certain additional peaks that can be attributed to (00l) lines with a higher *c* parameter value (c = 14.34 Å) prove that a doping reaction occurred in various parts of the compound. These results are in good agreement with NMR and electron diffraction studies that demonstrated a doped and non-doped phase, the latter of which indicates a 0.6 Å increase in the *c* parameter (as shown in Figure 4B), which is a value close to that found from XRD.

### 2.2. Raman Spectroscopy Analysis

**Figure 6:**
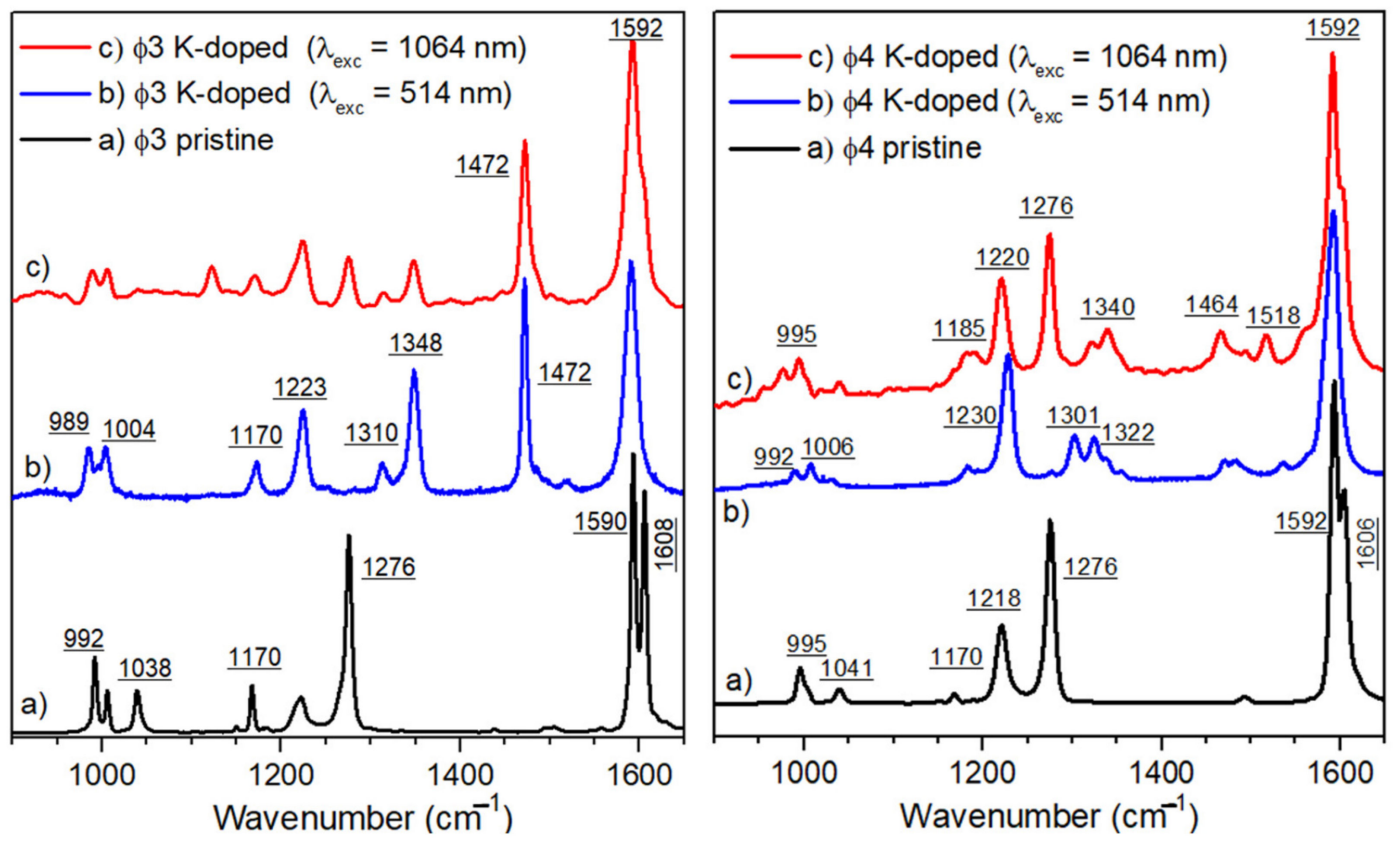
The Raman spectra of *p*-terphenyl (**left**) and *p*-quaterphenyl (**right**) is registered in the pristine (**a**) and reduced format 514.5 nm (**b**) and 1064 nm (**c**) excitation wavelengths.

It is well known that all features of Raman scattering spectra are signatures of the molecular structure. Therefore, all changes in the fingerprints of a given molecule can be considered as an indication of its evolution in its electronic and/or conformational structure. In Figure 6, the Raman scattering spectra of *p*-terphenyl and *p*-quaterphenyl are shown in the neutral (pristine) and doped forms. Concerning the pristine form, the attributions of vibrational modes (as listed in Table 2) were conducted with the support of previous publications [20,21,22,23,24,25,26]. The results presented in this table are self-explanatory; as such, the discussion is limited to the main bands, which showed pronounced evolutions during the reduction process.

The most characteristic features of the Raman spectra can be outlined as follows:

(i) Two strongly overlapping sharp peaks at 1595 and 1610 cm^−1^ that can be assigned to the aromatic C-C stretching deformations, which is consistent with the neutral state of the molecules (i.e., mono- and para-substituted benzene rings);
(ii) A band at 1288 cm^−1^ is usually attributed to the C-C interring stretching;
(iii) A weak band at ca. 1220 cm^−1^ together with a band at ca. 1170 cm^−1^ are characteristic of the C-H, when in plane bending deformations in the aromatic ring;
(iv) A band that is pointed at 996 cm^−1^, which is always absent in para-substituted benzene rings, confirms the coexistence of mono-substituted rings.

Certain additional bands can also be observed around 780 cm^−1^, in the 3000–3200 cm^−1^ range, which are assigned to ring deformations and aromatic (sp2) C-H stretching bands, respectively. It should be noted that no frequency dispersion was observed as a function of the excitation wavelength (at room temperature). Moreover, due to the presence of a center of symmetry in the two molecules, infrared absorption spectra exhibit inactive bands in Raman spectra (i.e., the rule of mutual exclusion). Of particular note were the four intense IR bands that were observed around 681, 750, 830, and 1479 cm^−1^ (Figure S5 in the Supplementary Materials). The first three bands are associated with “out of plane” C-H bending vibrations and the fourth is assigned to the ring C-C asymmetric stretching vibration. The complete interpretation of ir absorption spectra can be found in [21]. It should be emphasized here that only the IR absorption spectra of neutral form of molecules have been studied in this study. The organic molecules used here, doped with alkali metals, were unstable under atmospheric condition (the experimental conditions of our setup).

In order to follow the evolution of the characteristic bands upon the chemical reduction process, the vibrational data of the reduced molecules are compared to those of a pristine oligophenyl, as can be seen in Figure 6. First, an evolution of the double band at 1608–1590 cm^−1^ is observed, which is after the reduction appears as a broad overlapping band in the spectral range around 1592 cm^−1^. Second, the bands at 1280 cm^−1^, in the neutral forms, disappear. In addition, two or three components grow between 1300 and 1350 cm^−1^, with a maximum around 1340 cm^−1^. The characteristic aromatic bands at 1170–1220 cm^−1^ are slightly shifted to 1183 and 1227 cm^−1^. Moreover, the characteristic band of the mono-substituted ring is observable at 996 cm^−1^.

**Figure 7:**
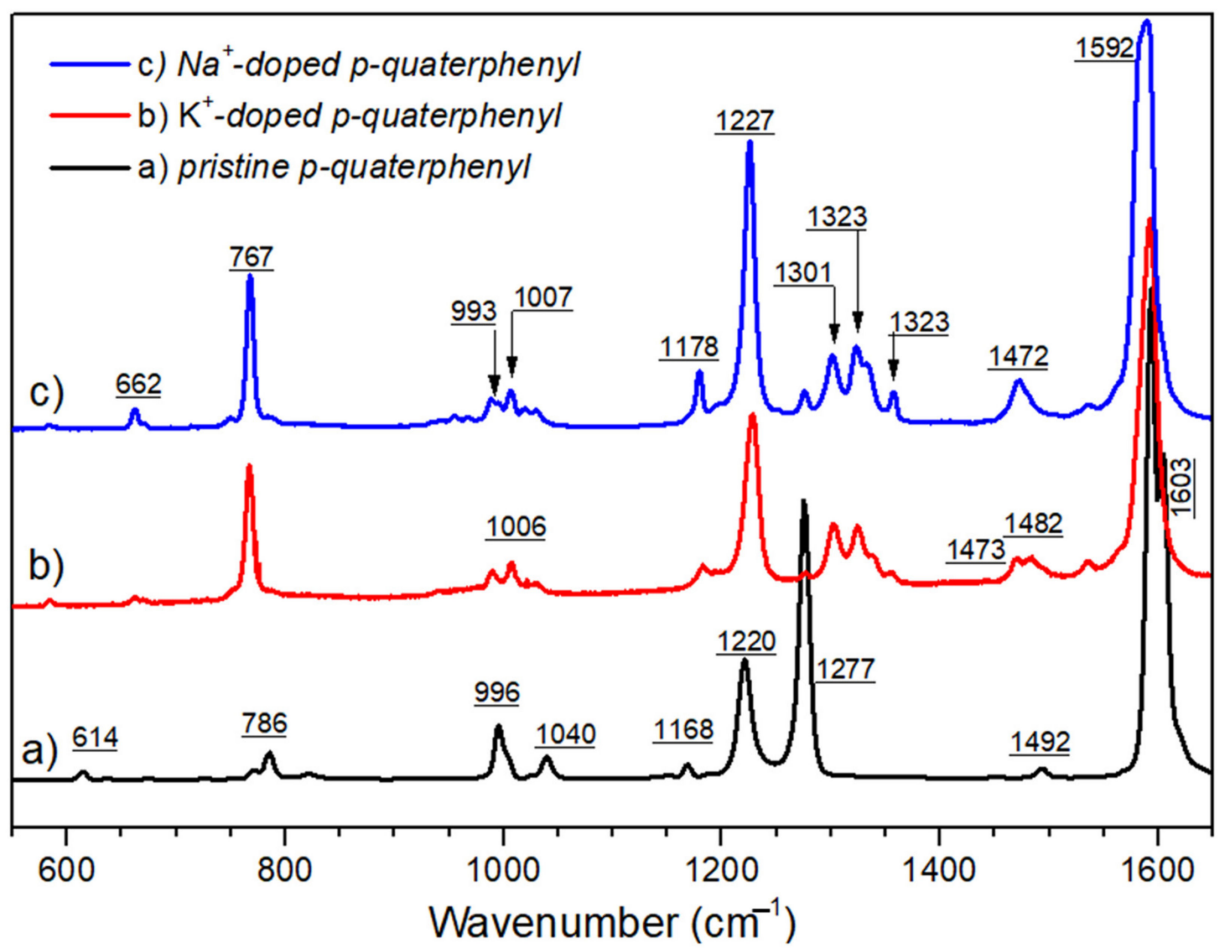
A comparison of Raman spectra of (**a**) pristine *p*-quaterphenyl, (**b**) K-doped, and (**c**) Na-doped quaterphenyl, with 514 nm as the excitation wavelength.

Figure 7 presents a comparison between Raman data that was observed between pristine *p*-quaterphenyl, K^+^-doped, and Na^+^-doped *p*-quaterphenyl. From the perspective of a careful analysis of Raman spectra, it is clear (as can be seen in Figure 7) that the evolutions of the spectra are small; further, it can be hypothesized from this observation that the doping processes with sodium or potassium are quite similar despite the difference in size of the doping ions.

However, the Raman data of *p*-terphenyl differ from that of *p*-quaterphenyl by the appearance of a strong band at 1472, at both 514.5 nm and 1064 nm as the excitation lines. From these observations it may be suggested that the emergence of radical anions in *p*-quaterphenyl does not exactly follow the same evolutions as in the case of *p*-terphenyl. At room temperature, it is now established than the neutral structure of *p*-terphenyl is monoclinic with the space group P_21/a_ and two molecules via unit cell. On consideration of this matter, the active Raman modes of crystal are mainly due to the combination of the vibrational Ag modes of individual molecules. In the first approximation, the molecules are considered planar, as such the point group is D_2h_. In the doped compound, the non-symmetrical distribution of the charge on the molecule can cause a break in symmetry. This allows the inactive modes of vibration to appear on the Raman spectrum.

**Scheme 1:**
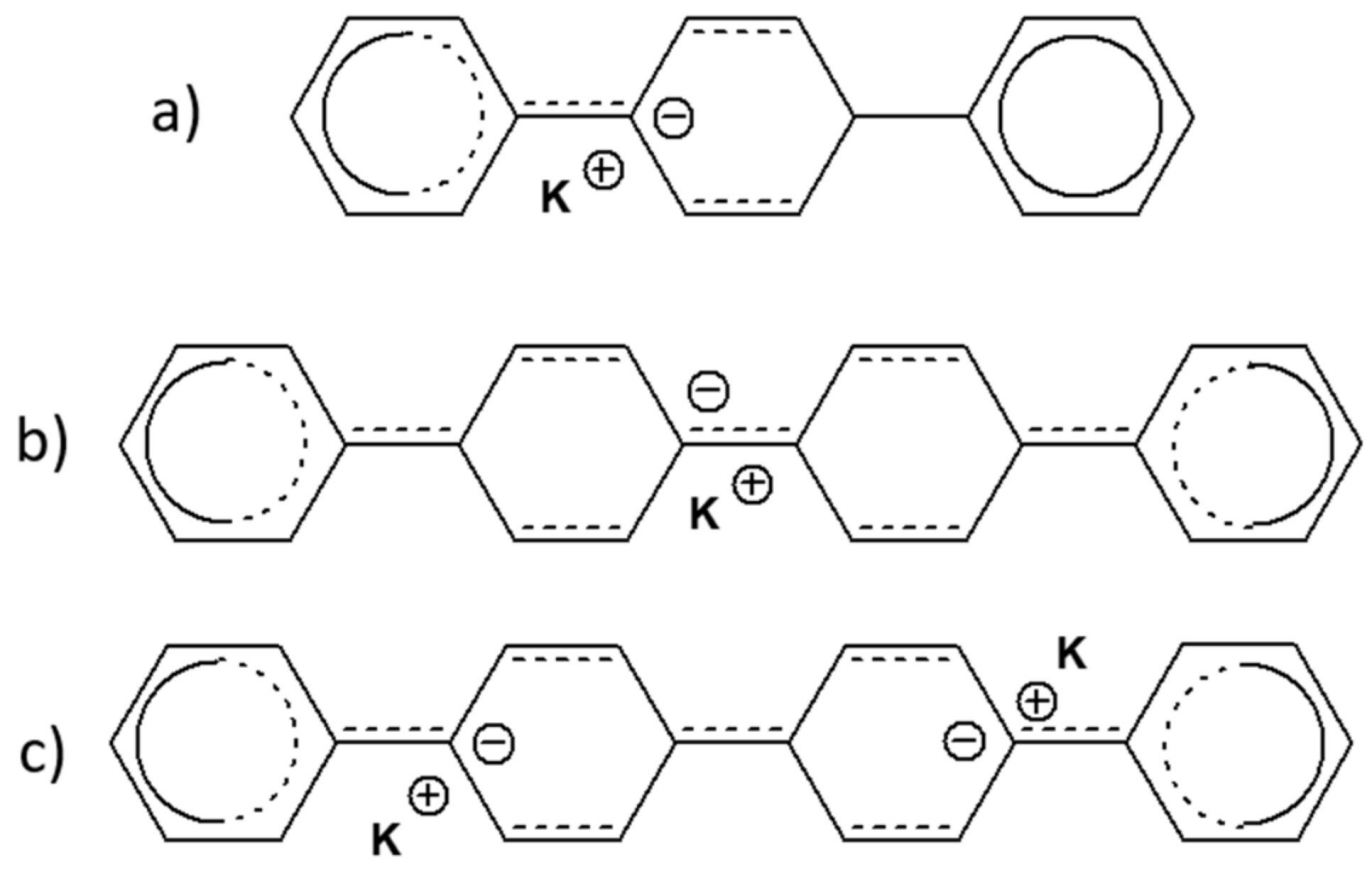
The chemical structures of (**a**) *p*-terphenyl reduced to radical anion (**b**) *p*-quaterphenyl reduced to radical anion, and (**c**) *p*-quaterphenyl reduced to dianion.

In order to interpret the appearance of the intense band at 1472 cm^−1^, in the doped *p*-terphenyl, it is assumed that its origin lies in the intense mode observed, via the infrared absorption spectroscopy, at 1478 cm^−1^. While this mode is Raman inactive (Bu Mode), its appearance could be made possible by the loss of symmetry in the molecule by a strong localization of the charge on a component of the molecule, as shown in Scheme 1a.

In regard to quaterphenyl, it can, therefore, be postulated that the charges are mainly delocalized on the two phenyl rings and that symmetry can, thus, be kept (Scheme 1b). Finally, in regard to the high doping level, there is a widening of the bands attributed to the C-C interring stretching bonds, which could indicate the presence of dianions (Scheme 1c).

## 3. Materials and Methods

### 3.1. Synthesis

The *p*-terphenyl (ϕ3) and *p*-quaterphenyl (ϕ4) used were purchased from commercial sources (Aldrich or Fluka, +99%), without further purification.

The doped materials were obtained using Na or K in order to promote the reduction that has been described in a previous work [27]. The used method, with liquid ammonia, resulted in a solid-state doped material, without the insertion of solvent. The obtained materials were annealed under vacuum for 36 h in an oven (at 120 and 130 °C for *p*-terphenyl and *p*-quaterphenyl, respectively). This process was performed in accordance with the procedure already described [27] in order to avoid, as much as possible, any non-uniform doping processes.

### 3.2. Electron Diffraction

Electron nanodiffraction studies were performed on a Hitachi HF2000 FEG-TEM, operating at 100 kV and 10 μA, whilst equipped with a Gatan CCD-camera. The samples were deposited on a holed carbon film supported by a copper grid, which was introduced into a Gatan Double tilt vacuum specimen holder.

Short adjustment and record times were used. This was performed due to the fact that pristine and doped PPP-oligomers were very sensitive to the electron beam. For each sample, many crystallites were studied in order to obtain a large number of diffraction patterns. In this manner, we were also able to overcome the problem of inhomogeneity. This was in addition to finding the possible existence of a phase that possesses a too small crystallite size to be detected in an X-ray pattern (please note, an electron diffraction wavelength is about 100 times smaller than that of X-rays and thus allows diffraction phenomena for smaller well crystallized particles). Therefore, the histogram of the measured cell parameters allowed us to determine their evolution upon doping. Having said that, these values were determined within a few per cent of error due to the difficult acquisition conditions of the patterns.

### 3.3. X-ray Diffraction

All the X-ray diffraction data collections were performed on an INEL CPS120-equipped powder diffractometer, which was set up in a horizontal Debye–Scherrer geometry. The CuK_α1_ radiation (λ = 1.540598 Å) was obtained with a bent quartz monochromator. The sample filled a 0.6 mm, in diameter, silica capillary. This technique allowed the use of a tiny amount of powder, which greatly aided with setting preferred orientation and moisture. In regard to this last purpose, the capillary was filled in an argon-atmosphere glove box (that was set at less than 1vpm H_2_O) and carefully closed with solvent-free glue. The classical Lindemann capillaries were not chosen due to their poor performances in terms of moisture protection. Indeed, they were chosen in spite of the fact that the use of silica leads to a higher absorption (i.e., the possible presence of bumps in the pattern low 2θ region). The as-prepared capillary was taken out of the glove box just before X-ray data collection.

When the quality of the data was good enough for the purposes of structure refinement (i.e., pristine material), Fullprof [28] software (version 2, Institut Laue-Langevin, France) was used for the Rietveld procedure, with use of the WinPLOTR [29] interface facility. In regard to the doped compounds, the poor quality of information concerning the new phase and the multi-phase nature of the pattern did not allow for the classical use of automatic indexing procedures, such as Treor [30] or Dicvol [31]. As such, the possible indexing of extra peaks was performed thanks to the Crystal Cracker [32] graphic indexing program. Subsequently, the cell parameters were refined using the Chekcell package [33].

### 3.4. Fourier Transform Infrared Spectroscopy (FTIR)

Fourier transform infrared spectra (FTIR) were recorded on a Bruker Vertex 70 spectrometer (4 cm^−1^), using an ATR Diamond accessory.

### 3.5. Raman Spectroscopy

The Raman spectra of neutral and oxidized molecules were recorded on a Renishaw InVia reflex spectrometer (λexc = 514.5 nm, laser power = 10 mW, 2 μm diameter spot, and typical exposure times 60 sec) or on a FT Raman Brucker RFS 100 spectrometer with the near-IR excitation line (1064 nm and laser power = 50 mW). All the experiments were conducted on crystalline powders, which were maintained under static secondary vacuum.

## 4. Conclusions

The heterogeneity of all the compounds included in this study were observed. A non-doped phase, as well as one or two doped phases were always found.

In regard to the K-doped terphenyl and K- or Na-doped quaterphenyl, only a *c* parameter variation was detectable. The possibility of a perfect constancy of all the cell parameters but c is difficult to imagine. Thus, it is more probable that, in regard to the pristine material, all these peaks found at the same location are due to a non-doped part of the compound. As such, the doped-phase would only be seen through information that was only coming from certain stacking differences along the c axis. In both oligomers, a potassium doping induces a *c* parameter increase. In regard to the K doped-quaterphenyl, a phase with a lower *c* parameter value was also observed. This doped phase is retrieved in Na-doped quaterphenyl (please note, in this compound there is no phase with a higher *c* parameter value). We can, thus, conclude that the quaterphenyl cell allows another site of doping when compared with the terphenyl cell, which can be occupied either by a potassium or by a sodium ion.

In relation to the Na-doped terphenyl, there is not only a *c* parameter variation, but also a drastic change in the *a* parameter (from 8.1041 Å in the pristine compound to 14.403 Å in the doped form). This can be due to polymerization, as found by NMR studies. Furthermore, this polymerization is difficult to locate for the Na-doped quaterphenyl; although, a second doped phase with only a space group change without atomic rearrangement was observed.

Complementary Raman spectroscopy investigations have shown that the reduction process is associated with the formation of radical anions or dianions that are located in different parts of the molecules. In the case of p-quaterphenyl, the radical anions and dianions created are delocalized on an entire conjugated unit; whereas in p-terphenyl, the negative charge seems more strongly confined on a small part of the conjugated molecule.

Finally, the authors hope that this work may contribute to the scientific community in aiding a better understanding of the doping of conjugated polymers. That is, in terms of the charge transfer process between the polymer chains and the counter-ions, whilst also allowing the optimization of the application of these systems.

## Figures and Tables

**Table 1:** The cell parameter refinement of Na-doped terphenyl.

hkl	2θ_obs_	2θ_calc_	2θ_obs_−2θ_calc_
002	9.89	9.91	−0.02
202	15.15	15.114	0.036
011	15.37	15.363	0.007
−103	16.02	16.018	0.002
012	17.66	17.622	0.038
301	17.95	17.917	0.033
302/004	19.85	19.853	−0.003
312	24.63	24.693	−0.063
−304	26.58	26.584	−0.004

**Table 2:** The main observed Raman frequencies (in cm^−1^) of *p*-terphenyl and *p*-quaterphenyl in neutral form, as well as when in the educed form by the potassium (K^+^) and sodium (Na^+^) ions (see also Figure S4 in the Supplementary Materials).

*p*-Terphenyl		*p*-Quaterphenyl				Benzene	
Raman Scattering (cm^−1^)		Raman Scattering (cm^−1^)					
Un-Doped	K-Doped	Na-Doped	Un-Doped	K-Doped	Na-Doped	Wilson Notation	Assignments
1608	1592	1592	1603	1592	1592	1596 (8a) 1596 (8b)	C-C stretch + C-H bend para benzene ring
1590			1596				
	1472	1482	1492	1473	1472	1486 (19a)	C-C stretch + C-H bend (intra ring)
							ring stretch + deformation
1276	1348	1350	1277	1340	1340		C-C stretch (inter ring)
	1310	1335		1301	1301		
1220	1223	1221	1220	1230	1227	1178 (9a)	Aromatic C-H bend (para benzene ring)
1170	1170	1190	1168	1180	1178		
1038	1004	1036	1040	1030	1040	1010 (12)	ring deformation (mono substituted ring)
1004	989	995	996	992	993		
992							
773	751	755	786	768	767	849 (10)	ring deformation

## Data Availability

Not available.

## Supplementary Materials

The following are available online at https://www.mdpi.com/article/10.3390/molecules27248699/s1, Figure S1: Rietveld refinement of terphenyl; Figure S2: Rietveld refinement of quaterphenyl; Figure S3: ϕ4-Na pattern compared with the pristine material; Figure S4: Infrared absorption spectra of pristine p-terphenyl and p-quaterphenyl; Figure S5: Raman spectrum of Na-doped, p-terterphenyl, with 514 nm as the excitation wavelength.

## Sample Availability

Not available.
