# Supplementary material for: The Influence of Alkali Metals on the Doping of Poly(p-phenylene) Oligomers
Source: Molecules. 2022 Dec 8;27(24):8699. doi: 10.3390/molecules27248699 (PMC9785341; doi:10.3390/molecules27248699)
Supplement: Supplementary file 1 [file molecules-27-08699-s001.zip › molecules-2048526-supplementary.pdf]

The influence of alkali metals on the doping of poly(*p*-phenylene) oligomers

Laura O. Péres [1], Rebeca Da Rocha Rodrigues [1,2] and Guy Louarn [2,*]

1 Laboratory of Hybrid Materials, Federal University of Sao Paulo, Diadema, SP, Brazil
2 Nantes Université, CNRS, Institut des Matériaux de Nantes Jean Rouxel, IMN, F-44000 Nantes, France
* Correspondence: guy.louarn@cnrs-imn.fr

Supporting Information

Figure S1. Rietveld refinement of terphenyl.

Figure S2. Rietveld refinement of quaterphenyl.

Figure S3: ϕ4-Na pattern (bottom) compared with the pristine material (top). Markers indicate $NaNH_2$ impurity.

Figure S4 : Infrared absorption spectra of pristine *p*-terphenyl and *p*-quaterphenyl.

Figure S5: Raman spectrum of Na-doped, *p*-terterphenyl, with 514 nm as the excitation wavelength.

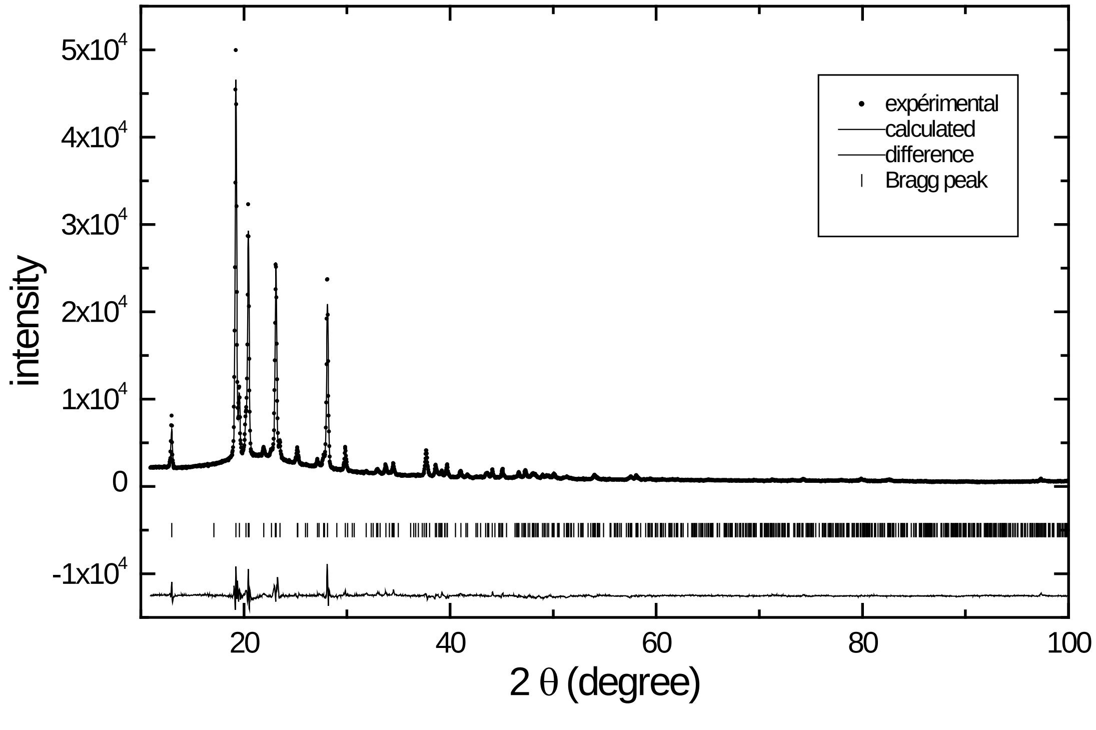

Figure S1. Rietveld refinement of terphenyl.

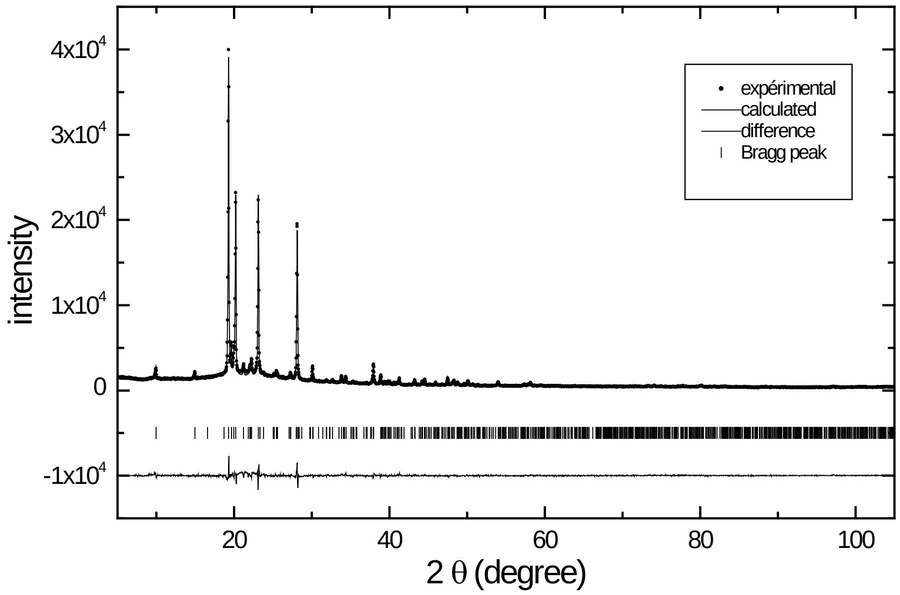

Figure S2. Rietveld refinement of quaterphenyl.

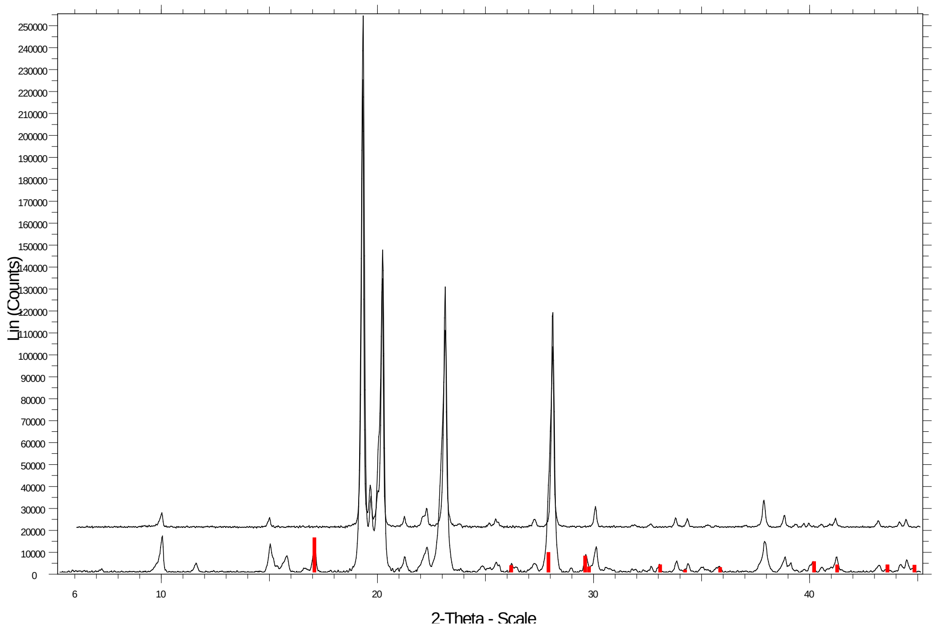

Figure S3: ϕ4-Na pattern (bottom) compared with the pristine material (top). Markers indicate $NaNH_2$ impurity.

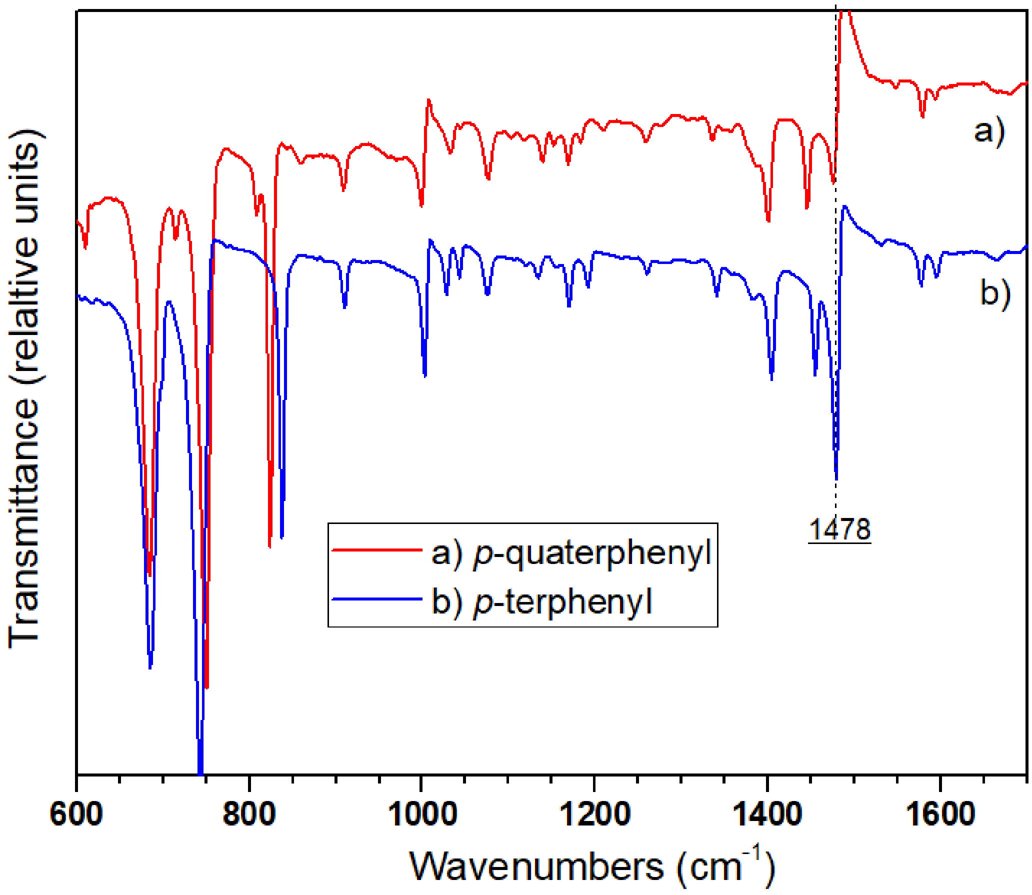

Figure S4 : Infrared absorption spectra of pristine *p*-terphenyl and *p*-quaterphenyl.

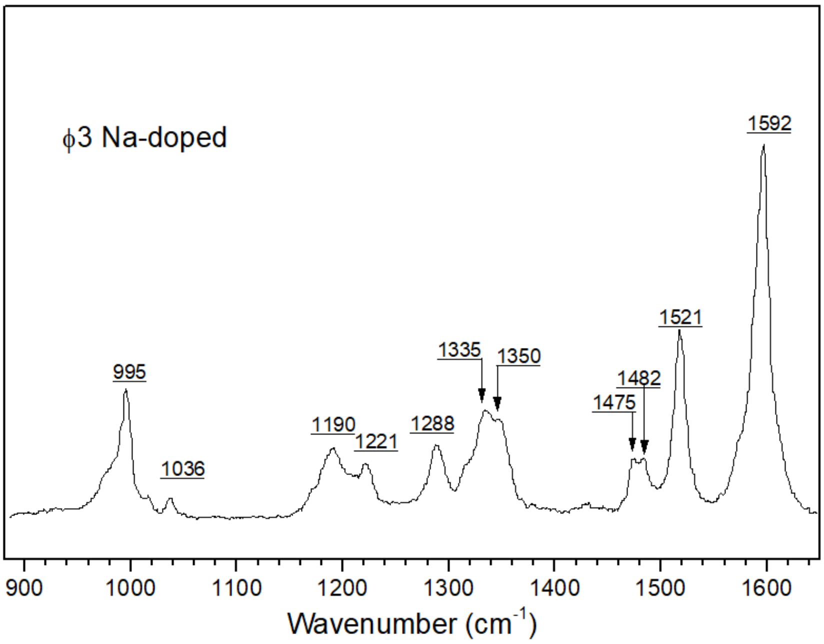

Figure S5: Raman spectrum of Na-doped *p*-terterphenyl, with 514 nm as the excitation wavelength.
